# The Charity Commissioners and the Lords Committee

**Published:** 1893-05-20

**Authors:** 


### THE CHARITY COMMISSIONERS AND THE LORDS COMMITTEE.

A return containing papers and correspondence between Lord Sandhurst and the Chief Charity Commissioner have recently been published. It deals with paragraph 567 of the Lords' Report on Metropolitan Hospitals, in which the Committee express regret "that by the action of the Charity Commissioners, St. Thomas's Hospital was prevented from using its own money." This statement the correspondence shows to be the exact opposite of the fact, and we think there is cause for regret that the Chairman of the Committee of the House of Lords, under all the circumstances, has not seen his way to frankly admit the error into which the Committee were led, and to facilitate the publication of the papers in question. The Charity Commissioners are not a too popular body, but they are entitled to justice, and it seems to us that they have had something less than justice in this business. We are glad that the Charity Commissioners have caused the return to be issued, and it may be usefully studied by the managers of all charities which have dealings with that body.

